# Removal and Recovery of Toxic Silver Ion Using Deep-Sea Bacterial Generated Biogenic Manganese Oxides

**DOI:** 10.1371/journal.pone.0081627

**Published:** 2013-12-02

**Authors:** Yuanjun Pei, Xiao Chen, Dandan Xiong, Shuijiao Liao, Gejiao Wang

**Affiliations:** 1 State Key Laboratory of Agricultural Microbiology, College of Life Science and Technology, Wuhan, P. R. of China; 2 State Key Laboratory of Agricultural Microbiology, College of Basic Sciences, Huazhong Agricultural University, Wuhan, P. R. of China; Glasgow University, United Kingdom

## Abstract

Products containing silver ion (Ag^+^) are widely used, leading to a large amount of Ag^+^-containing waste. The deep-sea manganese-oxidizing *bacterium Marinobacter* sp. MnI7-9 efficiently oxidizes Mn^2+^ to generate biogenic Mn oxide (BMO). The potential of BMO for recovering metal ions by adsorption has been investigated for some ions but not for Ag^+^. The main aim of this study was to develop effective methods for adsorbing and recovering Ag using BMO produced by *Marinobacter* sp. MnI7-9. In addition, the adsorption mechanism was determined using X-ray photoelectron spectroscopy analysis, specific surface area analysis, adsorption kinetics and thermodynamics. The results showed that BMO had a higher adsorption capacity for Ag^+^ compared to the chemical synthesized MnO_2_ (CMO). The isothermal absorption curves of BMO and CMO both fit the Langmuir model well and the maximum adsorption capacities at 28°C were 8.097 mmol/g and 0.787 mmol/g, for BMO and CMO, respectively. The change in enthalpy (ΔH^θ^) for BMO was 59.69 kJ/mol indicating that it acts primarily by chemical adsorption. The change in free energy (ΔG^θ^) for BMO was negative, which suggests that the adsorption occurs spontaneously. Ag^+^ adsorption by BMO was driven by entropy based on the positive ΔS^θ^ values. The Ag^+^ adsorption kinetics by BMO fit the pseudo-second order model and the apparent activation energy of E_a_ is 21.72 kJ/mol. X-ray photoelectron spectroscopy analysis showed that 15.29% Ag^+^ adsorbed by BMO was transferred to Ag(0) and meant that redox reaction had happened during the adsorption. Desorption using nitric acid and Na_2_S completely recovered the Ag. The results show that BMO produced by strain MnI7-9 has potential for bioremediation and reutilization of Ag^+^-containing waste.

## Introduction

Manganese (Mn) oxides are very useful for environmental remediation due to their adsorption, oxidation and catalysis activities. A variety of microorganisms, including bacteria and fungi, can oxidize Mn^2+^ to insoluble biogenic Mn oxide (BMO) that plays important roles in the biogeochemical cycle of Mn and also in controlling the distribution of metals and other trace elements in ocean and terrestrial environments [1]–[2]. Certain BMO (primarily δ-MnO_2_) showed much higher sorption and oxidation reactivity for a wide variety of metal ions compared to natural Mn oxides or chemically synthesized MnO_2_ (CMO) [3]–[4]. For example, BMO with todorokite-like crystal structure produced by *Leptothrix discophora* SP-6 exhibited a higher sorption capacity for metals than CMO [3]. The adsorption of Pb^2+^ by BMO produced by *Leptothrix discophora* SS-1 was 2–5 times greater than adsorption by CMO [5]. The adsorption of Co^2+^, Ni^2+^ and Zn^2+^ by BMO generated by the Mn-oxidizing fungus *Acremonium* sp. KR21-2 was nearly 10 times greater than adsorption by CMO (γ-MnO_2_) [6]. The Mn oxides generated by the deep sea strain *Brachybacterium* sp. Mn32 exhibited a capacity to adsorb Zn^2+^ or Ni^2+^ that was 2–3 times higher than that of freshly synthesized or commercially available MnO_2_ [1]. The successful adsorption of Cd^2+^, Fe^3+^, As^5+^, Cu^2+^ and Mn^2+^ by BMO has also been reported [7]–[10]. The effectiveness of BMO is mainly dependent upon their large specific surface area [5], [11], smaller grain size [12], and increased octahedral cavity structure [3], [13], which ensure that the adsorbed substance is incorporated into the crystal structure of the oxide [14]. However, small specific surface area was also reported for certain BMO [15]. Thus, the mechanism behind the high adsorption capacity of BMO is still disputed.

Products containing silver ions (Ag^+^) are widely used in electronics, electroplating, chemical synthesis, manufacture of photosensitive materials, leading to a large amount of silver-containing waste [16]–[17]. The removal and recovery of Ag^+^ is primarily accomplished through precipitation, electrolysis, adsorption, ion exchange and redox reactions [18]–[19]. Of these methods, the removal of Ag^+^ by adsorption is especially attractive because it uses less energy, generates less secondary pollution and is only weakly dependent on the silver structure [20]. Most Ag^+^ adsorption studies use chemical adsorbents [21]. New types of adsorbents such as chelating materials, activated carbon fiber, polymers with free amine groups and biogenic adsorbents have also been used [22]–[25]. In deep sea, silver exists mostly within sulfide deposits, which presents at about 1,400–3,700 m deep [26]. Adsorbent BMO could be generated from manganese-oxidizing microorganisms that are widespread in the environment [13]. To the best of our knowledge, the successful use of a BMO for Ag^+^ removal has not yet been reported. Furthermore, the mechanisms of adsorption by most BMOs are largely unknown.

In this study, we assessed the Ag^+^ adsorption and desorption capacity of BMO produced by the deep sea Mn-oxidizing bacterium *Marinobacter* sp. MnI7-9. The aims of this study were (1) to examine the Ag^+^ adsorption capacity of BMO and compare that to CMO, (2) to determine the optimal conditions for adsorption and identify the adsorption mechanism, and (3) to develop an effective method for recovering Ag.

## Materials and Methods

### Ethics Statement

No specific permissions were required for these locations/activities since it is a public Ocean and sample collection did not involve endangered or protected species.

### Preparation of the bacteria, BMO and CMO

The Mn(II)-oxidizing bacterial strain MnI7-9 was isolated from deep sea sediment from the middle of the Indian Ocean and its genome was sequenced [27]. The strain was cultured in liquid A medium at 28°C with or without 10 mM MnCl_2_, as described in Liao et al. [2]. When adding 10 mM MnCl_2_, BMO produced after 7 d was mainly in δ-MnO_2_ form and formed a cover on the surface of the bacterial cells [2]. Then, the bacteria alone or the bacteria and the associated BMO were collected and dried in a vacuum freeze dryer at −50°C for 24 h. Analytically pure γ-MnO_2_ (CMO) was purchased from Sinopharm Chemical Reagent Co., Ltd, China, to use as a control.

### Analysis of adsorption efficiency for Ag^+^ at different pH values

AgNO_3_ solutions (0.5 or 20 mmol/L) were adjusted with different pH values using HNO_3_ or NaOH and added to flasks containing 0.5 g of BMO or CMO. The flasks were stoppered and shaken at 150 rpm for 3 d at 28°C. Then, 1 mL of the mixture was removed from the flask and centrifuged. The Ag^+^ concentration in the supernatant was measured by an atomic absorption spectrometer (AAS) (986A, Beijing Puxi General Instrument Co., Beijing, China).

The adsorption percentage of Ag^+^ was calculated as follows:
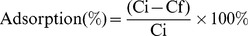
(1)where C_i_ and C_f_ are the initial and final Ag^+^ concentrations (mmol/L), respectively.

### Adsorption kinetics

To analyze the Ag^+^ adsorption kinetics, the 0.5 and 20 mmol/L AgNO_3_ solutions with the 0.5 g of BMO or CMO were shaken at 150 rpm for 3 d at 28°C, and 1 mL samples were taken at 1, 2, 3, 5, 7, 9, 10 and 22 h for the 0.5 mmol/L AgNO_3_ solution, and at 1, 2, 4, 8, 12, 22, 27, 36, 46, 60 and 72 h for the 20 mmol/L AgNO_3_ solution. The samples were centrifuged, and the Ag^+^ concentration in the supernatant was measured by AAS.

### Analysis of the Ag^+^ adsorption isotherm

To determine the Ag^+^ adsorption isotherm, different concentrations of AgNO_3_ (0.5 – 60 mmol/L) with the 0.5 g of BMO or CMO were incubated at 18, 28, 37 or 46°C.

The amount of Ag^+^ adsorbed per unit mass of adsorbent (q_e_) was calculated using the following equation:
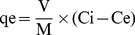
(2)where C_i_ and C_e_ represent the initial and equilibrium concentrations (mmol/L), respectively. V is the volume of the AgNO_3_ solution (L) and M is the mass of the adsorbent (g).

### Morphology, X-ray photoelectron spectroscopy (XPS) and specific surface area analysis

BMO with and without adsorbed Ag^+^ was prepared by centrifuging at 8,000 rpm for 5 min. The pellets were dried in a vacuum freeze dryer at −50°C for 24 h. The morphology was observed by scanning electron microscopy (SEM) as described previously [2].

X-ray photoelectron spectroscopy (XPS) analysis was performed using a VG Multilab2000 X-ray photoelectron spectrometer with an Mg Kα X-ray source (1,253.6 eV). The survey scans were collected using a fixed pass energy of 100 eV and an energy step size of 1.0 eV, while the narrow scans had a pass energy of 25 eV and an energy step size of 0.05 eV. The spectra were analyzed and the XPS peaks were identified using Avantage software of Thermo Electron Corporation. All spectra were charge-corrected to give the adventitious C (1 s) spectral component (C-C, C-H) a binding energy of 284.80 eV [28].

The specific surface area of the samples was determined by the N_2_ adsorption method (BET) with an F-Sorb3400 surface analyzer [29].

### Recovering Ag from BMO

After adsorption of Ag^+^ by BMO, 100 mL of a 100 mmol/L Na_2_S solution was added. The flasks were stoppered and shaken for 12 h at 28°C, centrifuged at 8,000 rpm for 5 min, and the precipitate was collected. The precipitate was then mixed with 65% nitric acid for 15 min in a 75°C water bath and centrifuged at 8,000 rpm for 5 min. The supernatant was mixed with 100 mmol/L NaCl in the dark for 30 min, and then centrifuged at 8,000 rpm for 5 min. The precipitate was collected and dried in a vacuum freeze dryer at −50°C for 24 h.

## Results and Discussion

### The Ag^+^ adsorption efficiencies of BMO and CMO

**Figure 1:**
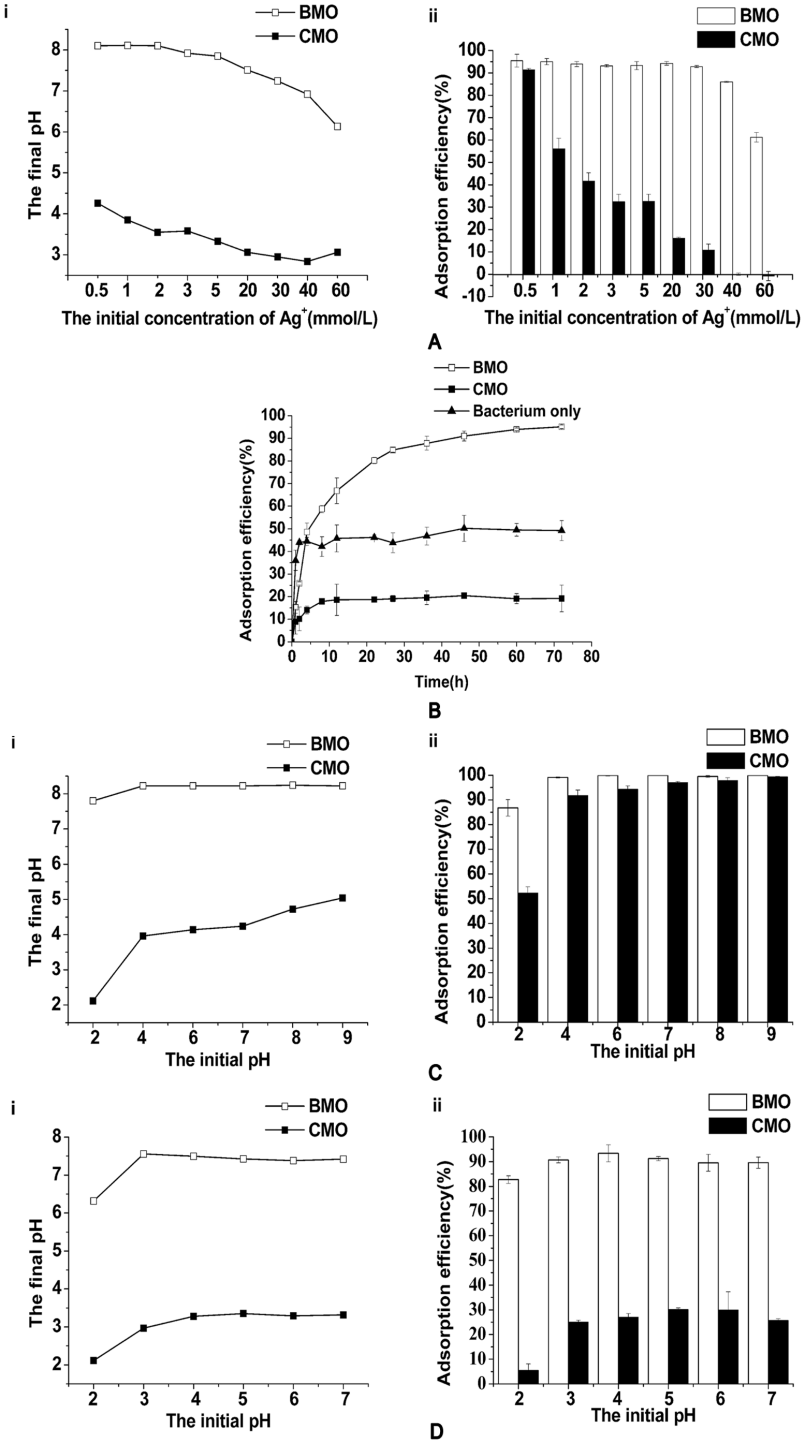
The influence of pH, Ag^+^ concentration and adsorbent type on Ag^+^ adsorption efficiency. (**A**), The pH values after the adsorption at different initial concentrations of Ag+ (i) and the adsorption efficiencies of Ag+ by BMO (□) and CMO (▪) at different initial concentrations of Ag+ (ii); (B), the adsorption efficiencies of 20 mmol/L Ag+ by BMO (□), CMO (▪) and the MnI7-9 bacterium only (▴); (C), The pH values after adsorption (i) and the adsorption efficiencies of 0.5 mmol/L Ag+ at different initial pH values (ii); (D), The pH values after adsorption (i) and the adsorption efficiencies of 20 mmol/L Ag+ at different initial pH values (ii).

The effects of the initial concentrations of Ag^+^ (0.5–60 mmol/L) on Ag^+^ adsorption efficiency at 28°C are shown in Figure 1A(ii). The adsorption efficiency of BMO was mainly greater than 95% at Ag^+^ concentrations of 0.5 to 20 mmol/L, while CMO adsorption efficiency was much lower, only approaching 95% at 0.5 mmol/L Ag^+^. The pH values after adsorption decreased as the initial Ag^+^ concentrations increased in Figure 1A(i). Taking the adsorption of the solvent molecule (water) into account, the adsorption efficiency of Ag^+^ by CMO was negative at Ag^+^ concentrations of 40 to 60 mmol/L (Figure 1A(ii)). These negative values will be discussed further below.

As shown in Figure 1 B, the adsorption efficiency of BMO was greater than 95%, while the adsorption efficiency of the bacteria alone without BMO was less than 50%. The adsorption efficiency of CMO was only approximately 20% at 20 mmol/L Ag^+^. Therefore, the adsorption efficiency of BMO is much higher than the adsorption efficiency of the other two adsorbents. The fact that the adsorption efficiency of the bacterial cells was only approximately 50% indicates that the bacterial cells made only a partial contribution to Ag^+^ adsorption by the bacteria and BMO together.

The adsorption efficiencies at various initial pH levels and the pH values after adsorption for 0.5 and 20 mmol/L solution are shown in Figure 1C and D, respectively. The Ag^+^ will precipitate from a 0.5 mmol/L solution at pH 10 and from a 20 mmol/L solution at pH 8 according to the rule of solubility product. Thus, the subsequent experiments were carried out at a pH range of 2–9 for 0.5 mmol/L Ag^+^ and 2–7 for 20 mmol/L Ag^+^. As shown in Figure 1C and D, the highest adsorption efficiency of BMO was approximately 95% at an Ag^+^ concentration of 0.5 and approximately 90% at an Ag^+^ concentration of 20 mmol/L. However, the highest adsorption efficiency of CMO was only approximately 25% from a 20 mmol/L Ag^+^ solution. When pH>4, the adsorption efficiency of both fluctuated around the highest adsorption efficiency of the present experiment conditions whatever the initial pH value was. The pH of BMO after adsorption was in general greater than 7 (Figure 1A, C and D), while the pH of CMO was around 4 which was much lower than that of BMO (Figure 1 A, C and D), indicating that the pH had a significant influence on the adsorption efficiency. This may be due to the capability of the two Mn oxides to maintain the appropriate pH values. It has been reported that low pH values can decrease the affinity of Mn oxides for Ag^+^ due to competitive adsorption of H^+^ [30]. Higher pH values are also beneficial for an increase in net negative surface charge leading to oxides' higher affinity for metal ions [31]. The ability of BMO to maintain high pH values may be caused by the presence of K^+^, Na^+^, Mg^2+^ and various organic groups on its surface [32]. The pH values after adsorption decreased with increasing initial concentrations of Ag^+^ for both BMO and CMO (Figure 1 A). A likely explanation for this observation is that any Ag^+^ left in the solution after adsorption will hydrolyze, leading to a decrease in OH^−^, particularly at high initial concentrations of Ag^+^.

To confirm that BMO has a significantly higher adsorptive capacity than CMO under the influence of the pH or the Ag^+^ concentration, a two-way ANOVA analysis considering interactions was performed using the SAS program. Our results show that (i) without adjusting the initial pH, the effects of the Ag^+^ concentration, the adsorbent type (BMO or CMO) and the interaction of these two factors all have a highly significant effect (***) on the adsorption efficiency (Table 1 and Figure 1 A); (ii) at an Ag^+^ concentration of 0.5 mmol/L, the initial pH, the adsorbent type and the interaction of these two factors also has a highly significant effect (***) on the adsorption efficiency (Table 1 and Figure 1 C); and (iii) at an Ag^+^ concentration of 20 mmol/L, the difference in adsorbent performance is highly significant (***), the influence of the initial pH is significant (*) and the interaction between the adsorbent type and the initial pH is not significant (Table 1 and Figure 1D). These results indicate that variations in the adsorption efficiency are mainly due to the adsorbent type at high concentrations of Ag^+^ (e.g., 20 mmol/L). In other words, BMO had a stronger adsorptive capacity than CMO in all three experiments. The effect of the initial pH is weaker at higher concentrations of Ag^+^.

**Table 1:** Two-way ANOVA analysis considering interaction using the SAS program, showing the influence of pH, Ag^+^ concentration and adsorbent type (BMO or CMO).

The influence of Ag^+^ concentration and adsorbent type at natural pH (5.3–5.8)						
Source	DF	Sum of Squares	Mean Square	F Value	Pr>F	
Adsorbent	1	3.041×10^4^	3.041×10^4^	8.054×10^3^	<0.0001	***
Concentration	8	3.732×10^4^	4. Coles665×10^3^	1.236×10^3^	<0.0001	***
Adsorbent×Concentration	8	1.380×10^4^	1.726×10^3^	4.571×10^2^	<0.0001	***
Error	36	1.359×10^2^	3.78			
Corrected Total	53	8.167×10^4^				

The influence of pH and adsorbent type at the Ag^+^ concentration of 0.5 mmol/L						
Source	DF	Sum of Squares	Mean Square	F Value	Pr>F	
Adsorbent	1	6.864×10^2^	6.864×10^2^	3.182×10^2^	<0.0001	***
pH	5	4.072×10^3^	8.145×10^2^	3.776×10^2^	<0.0001	***
Adsorbent×pH	5	1.236×10^3^	2.472×10^2^	1.146×10^2^	<0.0001	***
Error	24	51.77	5.450×10^2^			

The influence of pH and adsorbent type at the Ag^+^ concentration of 20 mmol/L						
Source	DF	Sum of Squares	Mean Square	F Value	Pr>F	
Adsorbent	1	3.872×10^4^	3.872×10^4^	5.689×10^2^	<0.0001	***
pH	5	1.189×10^3^	2.378×10^2^	3.49	0.02	*
Adsorbent×pH	5	2.937×10^2^	58.73	0.86	0.52	
Error	24	1.633×10^3^	68.06			

### Adsorption kinetics

**Figure 2:**
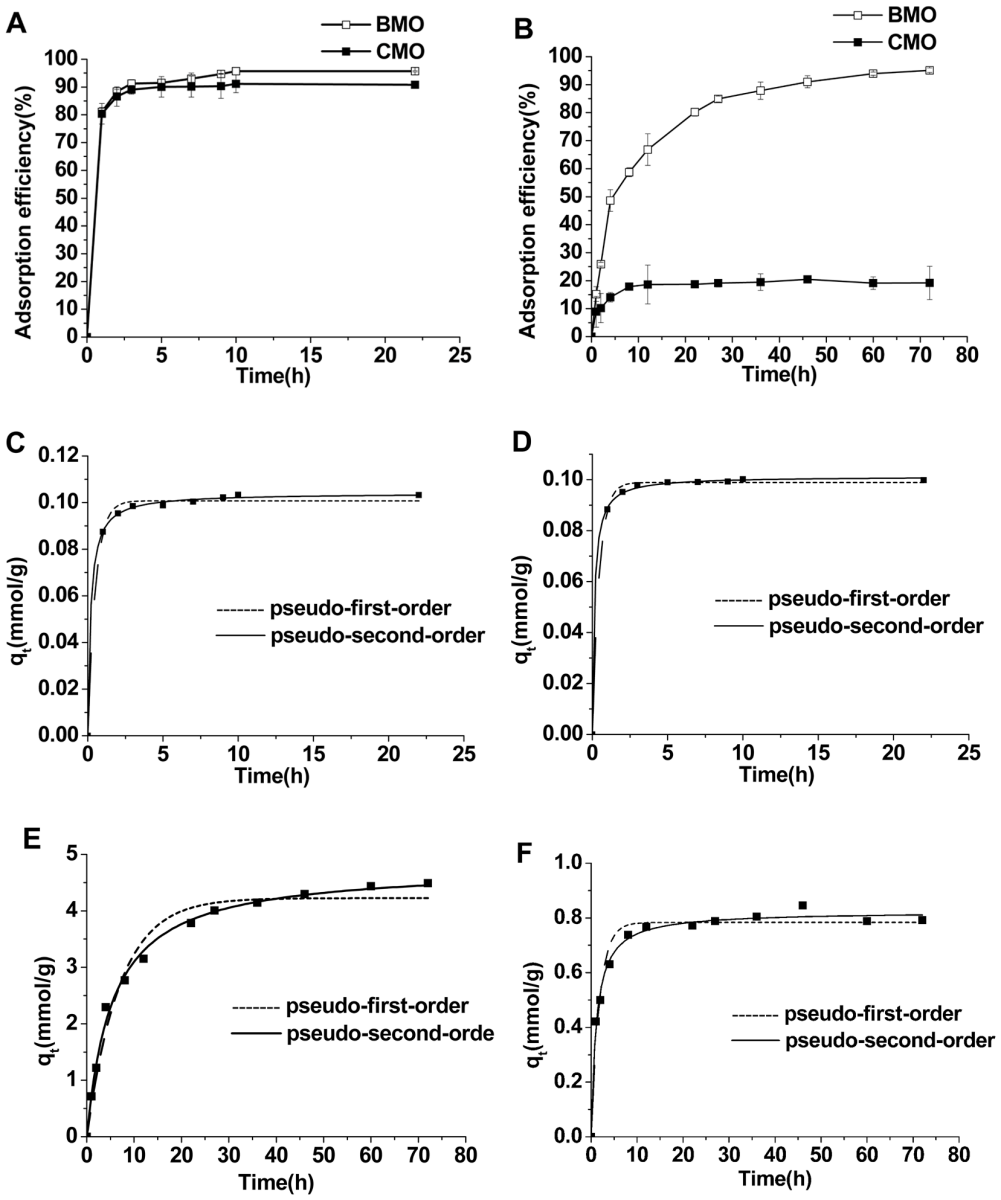
The Ag+ adsorption kinetics and fitting models. The adsorption kinetics of 0.5/L Ag+ (A) and 20 mmol/L Ag+ (B) by BMO (□) and CMO (▪). The pseudo-first order model and pseudo-second order model for adsorption of 0.5 mmol/L Ag+ by BMO (C) and CMO (D). The pseudo-first order model and pseudo-second-order model for adsorption of 20 mmol/L Ag+ by BMO (E) and CMO (F).

The adsorption kinetic analysis for BMO and CMO was performed using 0.5 and 20 mmol/L Ag^+^ solutions, representing low and high Ag^+^ concentrations, respectively. As shown in Figure 2, at high Ag^+^ concentrations, the adsorption process took longer to reach equilibrium. At both low and high Ag^+^ concentrations, the adsorption efficiency of BMO was above 95%; however, the adsorption efficiency of CMO was 90.86±0.60% and 19.18±5.92% at Ag^+^ concentrations of 0.5 and 20 mmol/L, respectively. The adsorption kinetic results confirmed that BMO performs better than CMO.

The pseudo-first order Lagergren rate equation was applied based on solid capacity. The equation is as follows:
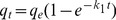
(3)where q_t_, q_e_ and k_1_ represent the adsorption quantity (mmol/g) at time t, the equilibrium adsorption quantity (mmol/g) and Lagergren rate constant (h^−1^), respectively [33]. We also used a pseudo-second order kinetic model based on solid phase sorption, as described in Ho and McKay [34]. The equation is as follows:
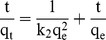
(4)


This equation can also be rearranged as described in [33]:
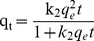
(5)where k_2_ is the pseudo-second order rate constant (gmmol^−1^h^−1^).

The results of non-linear fitting are shown in Figure 2. The constants of the different models, the residuals (Q) of non-linear fitting and the correlation coefficients (r^2^) at various concentrations are listed in Table 2.

**Table 2:** The parameters of Ag^+^ adsorption kinetics.

BMO						
Models	Constants	Concentrations (mmol/L)				
		0.5	10	20	60	100
Pseudo-first-order	k_1_ (h^−1^)	0.101	0.191	0.160	0.197	0.158
	q_e_ (mmol/g)	1.952	1.748	4.170	7.670	9.926
	Q	3.554×10^−5^	0.373	0.706	1.232	4.882
	r^2^	0.995	0.899	0.973	0.984	0.961
Pseudo-second-order	k_2_ (gmmol^−1^h^−1^)	50.99	0.117	0.042	0.032	0.019
	q_e_ (mmol/g)	0.104	1.956	4.726	8.441	11.11
	Q	4.979×10^−6^	0.182	0.202	0.497	1.419
	r^2^	0.999	0.951	0.992	0.993	0.989

CMO						
Models	Constants	Concentrations (mmol/L)				
		0.5	1	5	15	20
Pseudo-first-order	k_1_ (h^−1^)	2.181	0.469	0.376	0.488	0.561
	q_e_ (mmol/g)	0.099	0.119	0.157	0.556	0.781
	Q	1.005×10^−5^	7.974×10^−5^	0.001	0.019	0.016
	r^2^	0.999	0.993	0.946	0.937	0.973
Pseudo-second-order	k_2_ (gmmol^−1^h^−1^)	71.30	8.079	2.856	1.169	1.139
	q_e_ (mmol/g)	0.101	0.115	0.173	0.599	0.818
	Q	2.859×10^−6^	4.859×10^−5^	5.392×10^−4^	0.013	0.004
	r^2^	1.000	0.995	0.979	0.956	0.993

The adsorption kinetics of both BMO and CMO fit the pseudo-second order model better than the pseudo-first order model due to the lower Q value and the higher r^2^ values. Moreover, the higher the concentration of Ag^+^, the lower the value of k_2_ (Table 2). A pseudo-second order-type reaction is more likely to involve chemical adsorption (chemisorption) [34]. The r^2^ values for BMO were 0.995–0.899 for the pseudo-first order model and 0.999–0.951 for the pseudo-second order model, while the r^2^ values for CMO were 0.999–0.937 for the pseudo-first order model and 1–0.956 for the pseudo-second order model. The distinctly higher r^2^ values for BMO in the pseudo-second order model compared to the pseudo-first order model may indicate that the adsorption occurs primarily by chemisorption. In addition, the pseudo-second order model fits the experimental data better at low Ag^+^ concentrations compared to high Ag^+^ concentrations, which correlates well with previous results reported by Azizian [35]. Both models account for three rate-controlling steps, including film diffusion [36], intra-particle diffusion [37] and reaction [38].

The apparent activation energy of adsorption by BMO can be calculated by the Arrhenius equation:
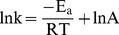
(6)where k, E_a_, R, T and A represent the apparent rate constant of the pseudo-second order model at a concentration of 20 mmol/L Ag^+^ at different temperatures, the activation energy (J/mol), the universal gas constant (8.314 J/mol K), temperature (K) and the pre-exponential factor, respectively. The value of – E_a_/R is equal to the slope of the straight line obtained by plotting lnk versus 1/T. The value of E_a_ is 21.72 kJ/mol, and the apparent rate constant increases as the temperature increases (Figure S1 in Supporting Information files).

### The adsorption isotherm

**Figure 3:**
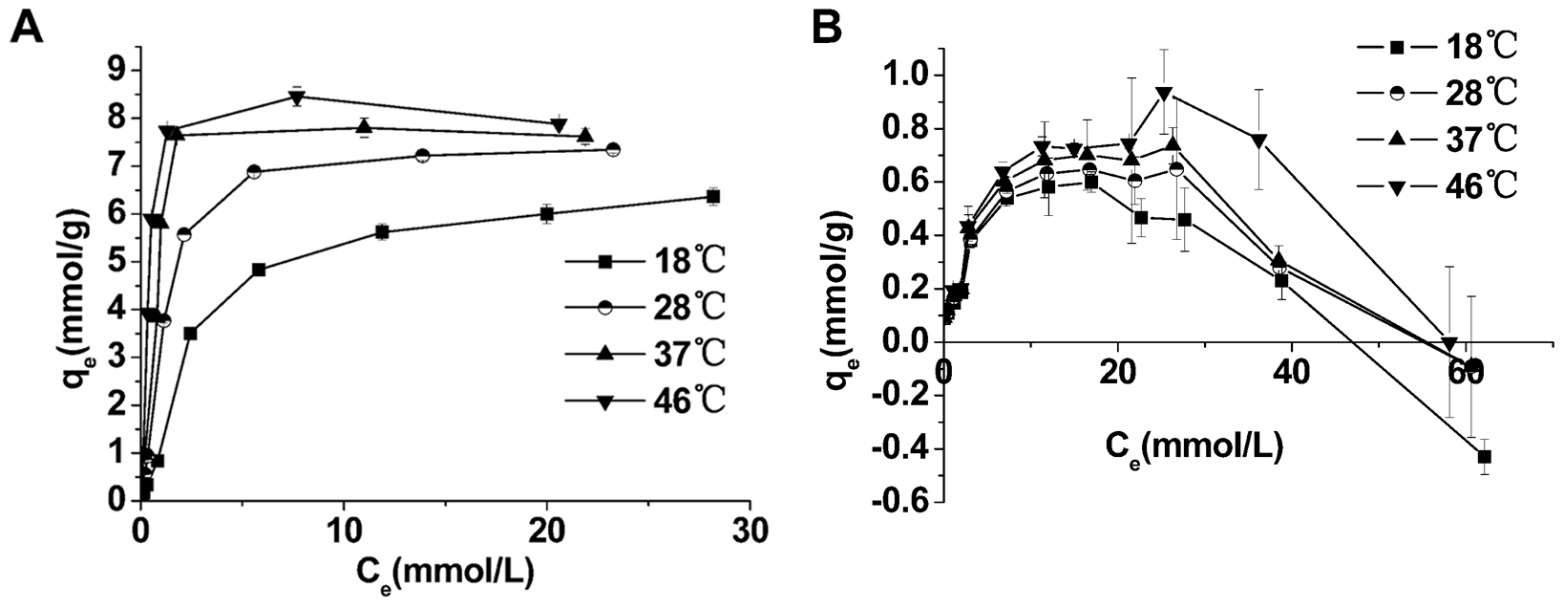
The Ag^+^ adsorption isotherms at different temperatures for BMO (A) and CMO (B).

The isothermal absorption curves of the two adsorbents are shown in Figure 3. The adsorption quantity tested in this experiment is the apparent adsorption quantity, due to the adsorption of other components in the solution such as water and NO_3_^−^. All of the isotherms exhibited maximum adsorption, and higher temperatures corresponded to higher maximum adsorption capacity. CMO isotherm resembles S-shaped excess isotherms due to the inclusion of negative values [39] (Figure 3B). Langmuir, Freundlich and Temkin models are often used to describe adsorption in binary solutions. In this study, the initial Ag^+^ concentration ranged from 0–60 mmol/L and 0–20 mmol/L for BMO and CMO, respectively, because the adsorption capacity of BMO and CMO began to decline at 60 and 25 mmol/L, respectively.

Each data point affects the slope and intercept of the straight lines when using the Langmuir equation. To avoid the significant bias caused by dilute concentrations, we used the Langmuir equation in linear form as follows [22], [33]:
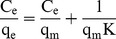
(7)where q_e_, q_m_ and *K* represent the equilibrium adsorption quantity (mmol/g) corresponding to Ag^+^ concentration C_e_ (mmol/L), the maximum adsorption quantity of Ag^+^ (mmol/g) and the Langmuir adsorption constant (L/mmol), respectively.

The Freundlich model is widely used to model chemical and physical adsorption. The linear equation is as follows [33], [40]:
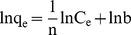
(8)where n is the constant related to the adsorption affinity of the metal ion and b is the constant at a particular temperature.

The Temkin model, which can be applied to chemical or monolayer physical adsorption, is calculated as follows [40]:
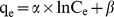
(9)where α and β are constants determined by the heat of the initial adsorption.

**Figure 4:**
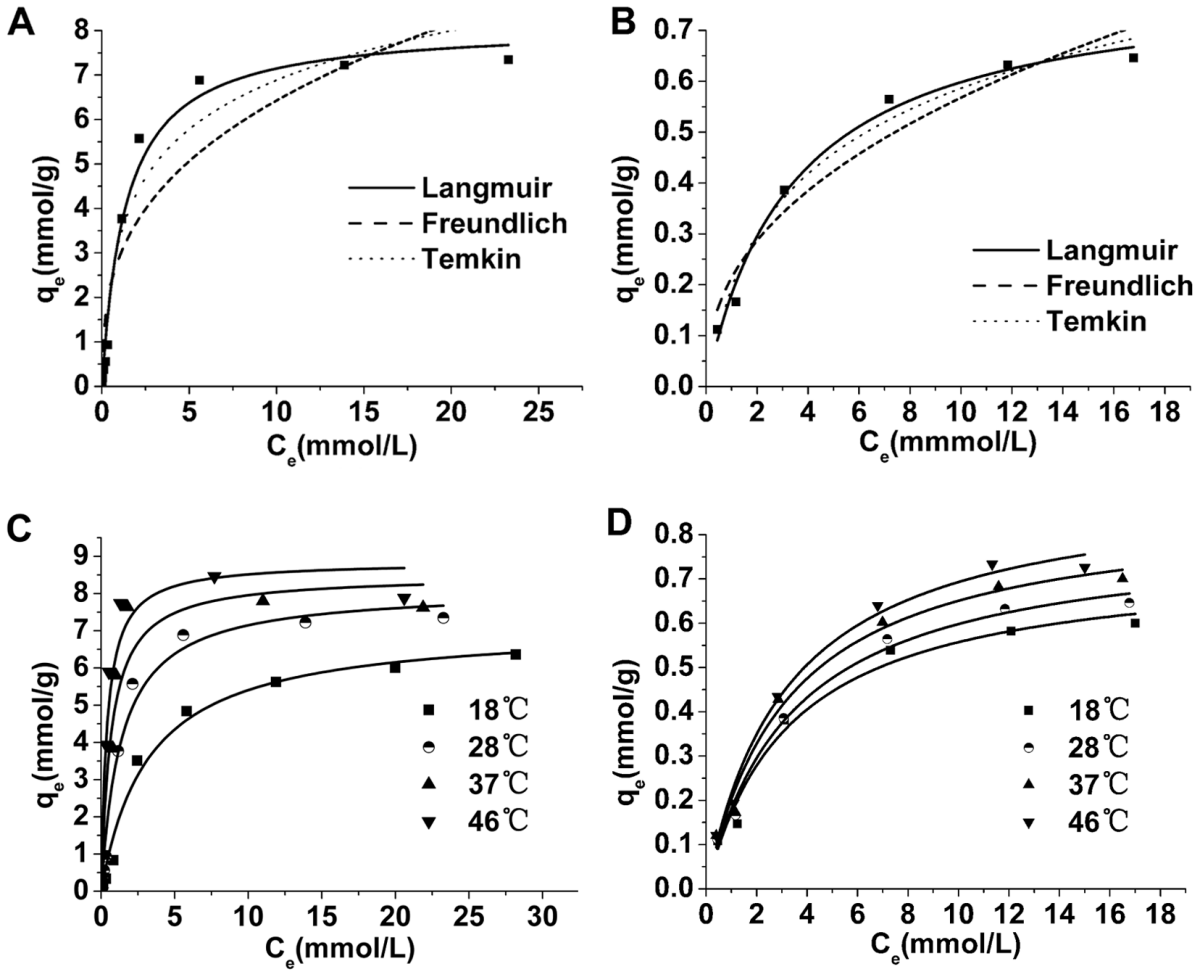
The fitting lines of isothermal models of Ag^+^ adsorption. The fitting lines of different isothermal models at 28°C for BMO (**A**) and CMO (**B**), and the Langmuir model at different temperatures for BMO (**C**) and CMO (**D**).

The values of the parameters and the correlation coefficients (r^2^) for the different isotherms at 18°C, 28°C, 37°C and 46°C are listed in Table 3. The Langmuir model is the best fit for the isotherms of both BMO and CMO because it has the highest r^2^ value. The intuitive fitness of each model is shown in Figure 4.

**Table 3:** The isotherm parameters of Ag^+^ adsorption.

Isotherm model		BMO				CMO			
		18°C	28°C	37°C	46°C	18°C	28°C	37°C	46°C
Langmuir	q_m_ (mmol/g)	7.669	8.097	8.104	8.237	0.733	0.787	0.850	0.898
	*K* (L/mmol)	0.193	0.522	1.004	1.677	0.299	0.306	0.315	0.325
	r^2^	0.980	0.993	0.994	0.994	0.982	0.990	0.986	0.990
Frenudlich	n	1.361	1.483	1.545	1.608	1.909	1.922	1.937	1.891
	b	0.881	0.581	2.542	3.281	0.162	0.176	0.194	0.205
	r^2^	0.953	0.931	0.864	0.805	0.940	0.962	0.950	0.967
Temkin	α	1.166	1.289	1.338	1.376	0.154	0.164	0.175	0.187
	β	2.399	3.702	4.588	5.302	0.190	0.207	0.233	0.248
	r^2^	0.935	0.923	0.884	0.880	0.958	0.970	0.964	0.972

The degree of favorableness of BMO and CMO for Ag^+^ was estimated using a dimensionless separation factor (the equilibrium parameter R) that indicates the likelihood that the adsorption process will proceed (R>1: Unfavorable; R = 1: Linear; 0<R<1: Favorable; R = 0: Irreversible). R is defined by the following equation [22], [33]:
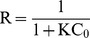
(10)where *K* is the Langmuir adsorption constant (L/mmol) and C_0_ is the initial concentration of Ag^+^ (mmol/L). The values of R for BMO and CMO were calculated as 0.031∼0.793 and 0.140–0.867, respectively. Hence, the adsorption of Ag^+^ onto both oxides is favorable.

The results shown in Table 3 and Figure 4 demonstrate that the Langmuir equation is the most appropriate model for these data. Using the values of *K* (Table 3) with various temperatures in the Langmuir equation, a series of thermodynamic parameters can be calculated according to van’t Hoff equation [22], [33], [40]:
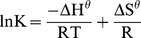
(11)where ΔH^θ^, ΔS^θ^, R, T represent enthalpy change (J/mol), entropy change (J/mol), the universal gas constant (8.314 J/mol K) and the temperature (K), respectively. The plot showing ln*K* versus 1/T is based on the data shown in Table 3. The slope and the intercept of the straight line are equal to −ΔH^θ^/R and ΔS^θ^/R, respectively. The Gibbs free energy change of adsorption (ΔG^θ^) can be calculated using the following equation [22], [33], [40]:
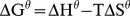
(12)


The linear relationship between ln*K* and 1/T is shown in Figure S2, and the values of ΔH^θ^, ΔS^θ^ and ΔG^θ^ are listed in Table 4.

**Table 4:** Enthalpy change, entropy change and Gibbs free energy of Ag^+^ adsorption on BMO and CMO.

MnO type	ΔG^θ^(kJ/mol)				ΔH^θ^(kJ/mol)	ΔS^θ^(kJ/mol)
	18°C	28°C	37°C	46°C		
BMO	−12.93	−15.42	−17.67	−19.91	59.69	0.249
CMO	−13.79	−14.35	−14.84	−15.34	2.287	0.055

The ΔG^θ^ was negative for both BMO and the CMO, indicating that Ag^+^ adsorption could occur spontaneously. All of the ΔG^θ^ values decreased as the temperature increased, which suggests that higher temperatures are beneficial for adsorption. This is in accordance with our experimental results. In addition, the lower ΔG^θ^ value for BMO implies that BMO has a stronger affinity for Ag^+^ than the CMO, which may be due to chemical bonds, van der Waals force or static adsorption.

The enthalpy change (ΔH^θ^) for BMO and CMO were 59.69 and 2.287 kJ/mol, respectively (Table 4). Both values were positive, indicating that the adsorption process is endothermic, which is in agreement with our analysis of the ΔG^θ^ values. The ΔH^θ^ of BMO was nearly 30 times higher than that of CMO. Most studies report that the ΔH^θ^ (integral heat of adsorption) of typical physical adsorption is close to the heat of liquefaction (0–20 kJ/mol), whereas the ΔH^θ^ of typical chemical adsorption is close to the heat of reaction (80–400 kJ/mol) and that both physical and chemical adsorption usually occur together in solution [41]. Our results suggest that CMO primarily acts by physical adsorption, while BMO primarily acts by chemical adsorption.

The enthalpy changes (ΔS^θ^) of BMO and CMO were 0.249 and 0.055 J/mol K, respectively (Table 4), indicating that the adsorption led to an increase in entropy. On one hand, the adsorption of Ag^+^ on adsorbent led to a decrease in entropy. On the other hand, the desorption of the substances (such as Mn^2+^) on adsorbent going with the adsorption leads to an increase in entropy. Therefore, the total entropy is a positive value, due to the complexity of adsorption in the solution [42]. The higher ΔS^θ^ value of BMO (Table 4) may be caused by the liberation of low molecular substances during Ag^+^ adsorption and the production of low molecular substances with the generation of Ag(0).

### The mechanism of adsorption

The SEM images in Figure S3 show clear differences at different Ag^+^ concentrations. Without the deposition of Ag^+^, BMO exhibited shuttle shapes (Figure S3 A). After adsorbing Ag^+^, BMO grains were noticeably larger and were covered by flocculi (Figure S3 B and C). This may be due to a change in electrostatic attraction among the grains when Ag^+^ was deposited on the surface [43]:

Ag^+^ + OH^−^ ↔ AgOH, and 2AgOH ↔ Ag_2_O + H_2_O.

**Figure 5:**
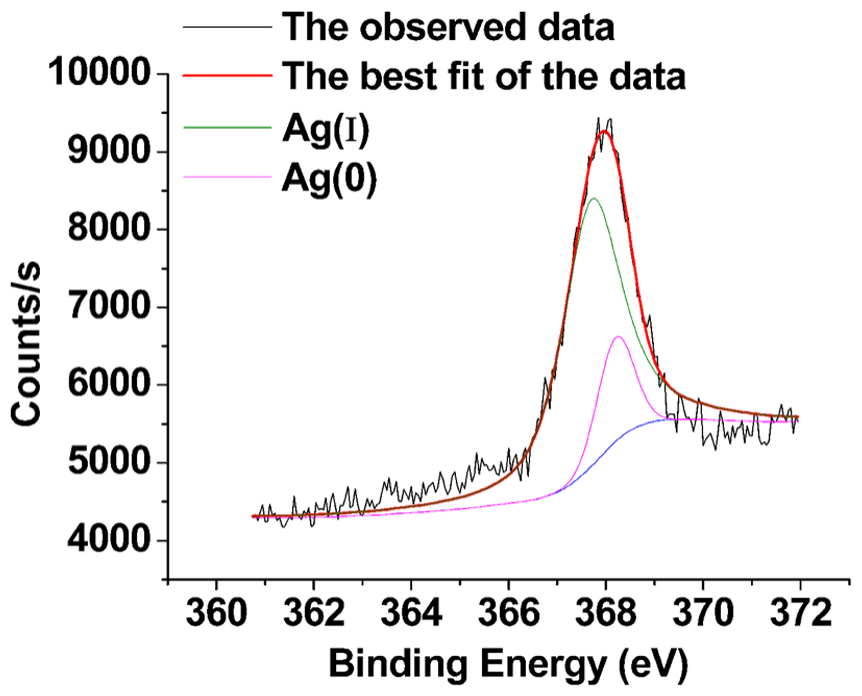
The XPS spectra of Ag (3d_5/2_) of BMO after absorbing Ag^+^. The upper black curve represents the observed data. The red curve is the best fit model for the data. The green curve represents the Ag(I) peak, while the pink curve is the Ag(0) peak.

The XPS results for Ag (3d_5/2_) are shown in Figure 5. In many studies, the Ag(Ι) peak is at 367.7 eV [44], while the Ag(0) peak is at 368.20 eV [45]. To determine the relative quantity of Ag(0) and Ag(Ι) on the surface of BMO after adsorption, we analyzed the Ag (3d_5/2_) spectrum. By calculating the peak areas in Figure 5, we determined that, after adsorption, the percentages of Ag(0) and Ag(Ι) on the surface of BMO were 15.29% and 84.71%, respectively. The results from the XPS analysis also suggest that adsorption by BMO involves a chemical reaction, as a small amount of Ag(Ι) was reduced to Ag(0) on the surface during adsorption (Figure 5). In this study, BMO produced by strain MnI7-9 was located on the surface of the bacteria [2], which means it is likely associated with a variety of reducing substances generated by the cells. It has been reported that Au^3+^, Pd^2+^, Pt^4+^ and Rh^3+^ can be reduced by functional groups released by dead organisms, such as the hydroxyl groups from glucose residues [46]–[48]. Dead *Bacillus licheniformis* R08 and *Lactobacillus* sp. A09 can reduce Ag(Ι) [46], [49]. Naik et al. used a phage surface display method to identify a polypeptide chain that can reduce Ag(Ι) [50]. The reduction of Ag(Ι) to Ag(0) detected by XPS analysis supports the involvement of a chemical reaction, which was also suggested by our thermodynamic analyses.

The specific surface areas of BMO and CMO were 0.473 m^2^/g and 17.32 m^2^/g, respectively. The smaller specific surface area of BMO suggests that surface area is not a major factor contributing to high adsorption capacity. A previous study had suggested that the larger specific surface area of BMO contributed to their high capacity for ion adsorption [1]. However, other studies showed that the adsorption capability is not always positively correlated with the size of the specific surface area, especially for biosorbents [15], [51]. For example, Chun et al. found that chars with smaller specific surface area (<200 m^2^/g) exhibited higher sorption of benzene than chars with greater specific surface area (>300 m^2^/g). In addition, BMO generated by *Bacillus* sp. WH4 has a strong adsorption capacity of Cd but lower specific surface area than the todorokite and some manganese oxides reported [15]. It may be due to the sorption by CMO occurring almost exclusively by surface adsorption but the sorption by BMO resulting from the surface adsorption and the concurrent smaller partition into the residual organic-matter phase.

### Recovering Ag from BMO

After 100 mL of a 20 mmol/L Ag^+^ solution was adsorbed by BMO, Ag was recovered and 0.29 g AgCl was obtained. The silver after adsorption by BMO occurred in the form of Ag (I) and Ag (0). Since the solubility of Ag_2_S is very low in water, we first added Na_2_S to BMO adsorbed Ag^+^ to convert various forms of silver to Ag_2_S. We used 65% nitric acid to dissolve Ag_2_S and Ag since they was not soluble in dilute nitric acid. Finally, the Ag^+^ solution was reclaimed through the formation of AgCl by adding excess Cl^−^. The 0.29 g AgCl contained 0.22 g of Ag, which was equal to the weight of Ag of 100 mL of a 20 mmol/L Ag^+^ solution. Thus, the recovery rate of Ag was 100%.

## Conclusions

This study provides the first successful demonstration of Ag^+^ removal using bacterial BMO, determined the mechanism of Ag^+^ adsorption by BMO and established a method for recovering the Ag. BMO has a stronger adsorptive capacity than the bacterium that produces it and CMO, which may be due to the ability of BMO to maintain high pH values in solution, as well as the presence of reducing substances on the surface of the oxide. The adsorption process by both BMO and CMO is best described by a pseudo-second order model. The isothermal absorption curves of BMO and CMO both fit the Langmuir model, as the maximum adsorption capacities and parameters were successfully calculated using this model. The maximum adsorption capacity of BMO was approximately 10 times higher than that of CMO. Both adsorption reactions are endothermic (ΔH^θ^>0), occur spontaneously (ΔG^θ^<0) and are driven by entropy (ΔS^θ^>0). The ΔH^θ^ value suggests that multiple mechanisms are involved in the adsorption process. BMO acts primarily by chemical adsorption, while CMO is more likely to act by a physical adsorption process. Finally, Na_2_S and 65% nitric acid are effective desorbents, yielding an Ag recovery rate of close to 100%. BMO produced by strain MnI7-9 shows potential applications for the bioremediation and reutilization of Ag^+^-containing waste.

## Supporting Information

Figure S1 **Plot of lnk vs. 1/T during Ag+ adsorption on BMO.**
(TIF)

Figure S2 **Plot of ln** ***K*** **vs. 1/T during Ag^+^ adsorption on BMO (A) and CMO (B).**
(TIF)

Figure S3 **The SEM images of BMO.** The SEM images of BMO without adsorbed Ag^+^ (**A**), after adsorbing 20 mmol/L Ag^+^ (**B**), and after adsorbing 100 mmol/L Ag^+^ (**C**).
(TIF)
