# Supplementary figures and images for: Removal and Recovery of Toxic Silver Ion Using Deep-Sea Bacterial Generated Biogenic Manganese Oxides
Source: PLoS One. 2013 Dec 2;8(12):e81627. doi: 10.1371/journal.pone.0081627 (PMC3847083; doi:10.1371/journal.pone.0081627)

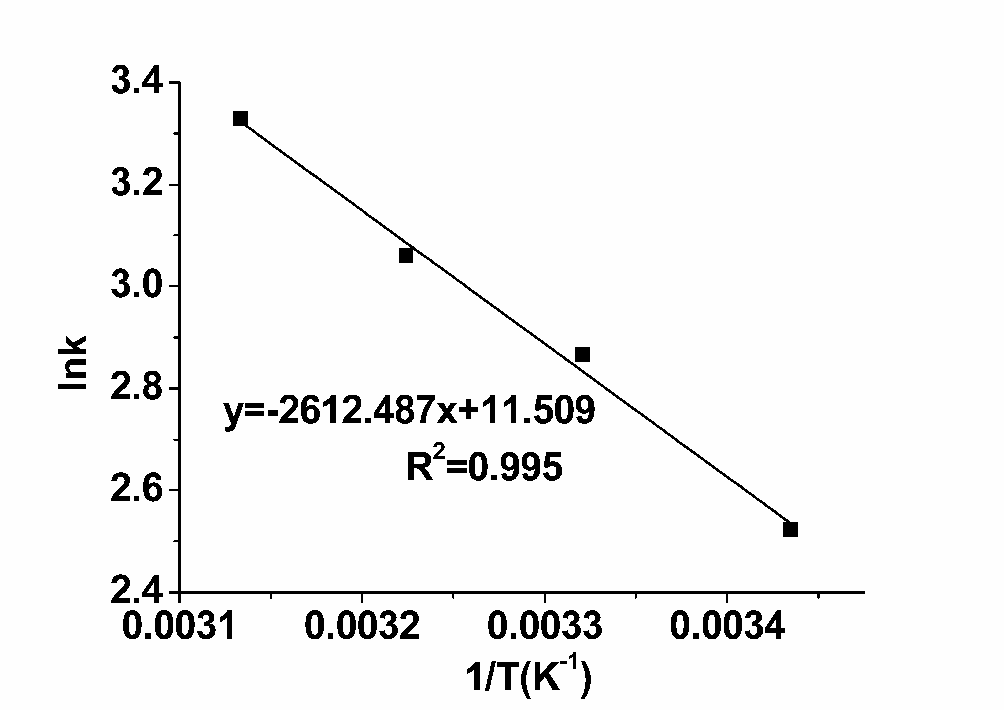

Supplement: Figure S1 — Plot of lnk vs. 1/T during Ag+ adsorption on BMO. (TIF) [file pone.0081627.s001.tif]

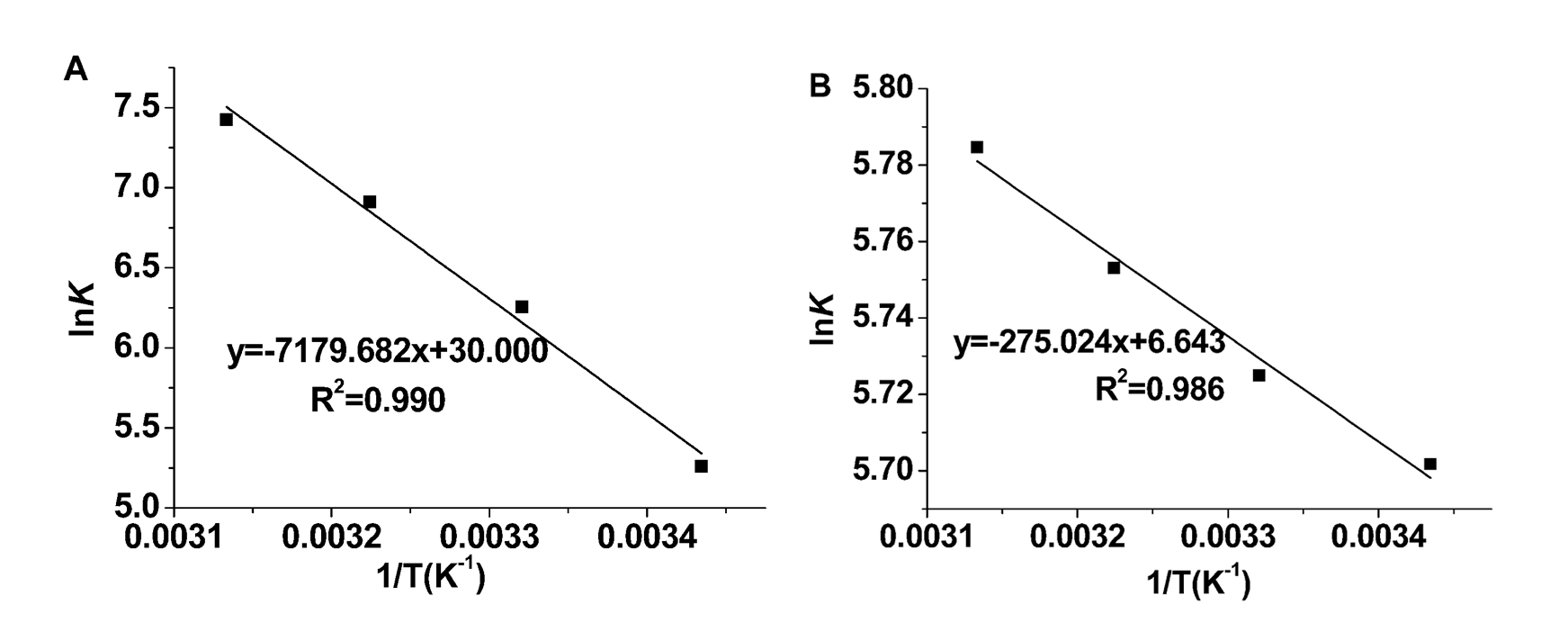

Supplement: Figure S2 — Plot of ln K vs. 1/T during Ag+ adsorption on BMO (A) and CMO (B). (TIF) [file pone.0081627.s002.tif]

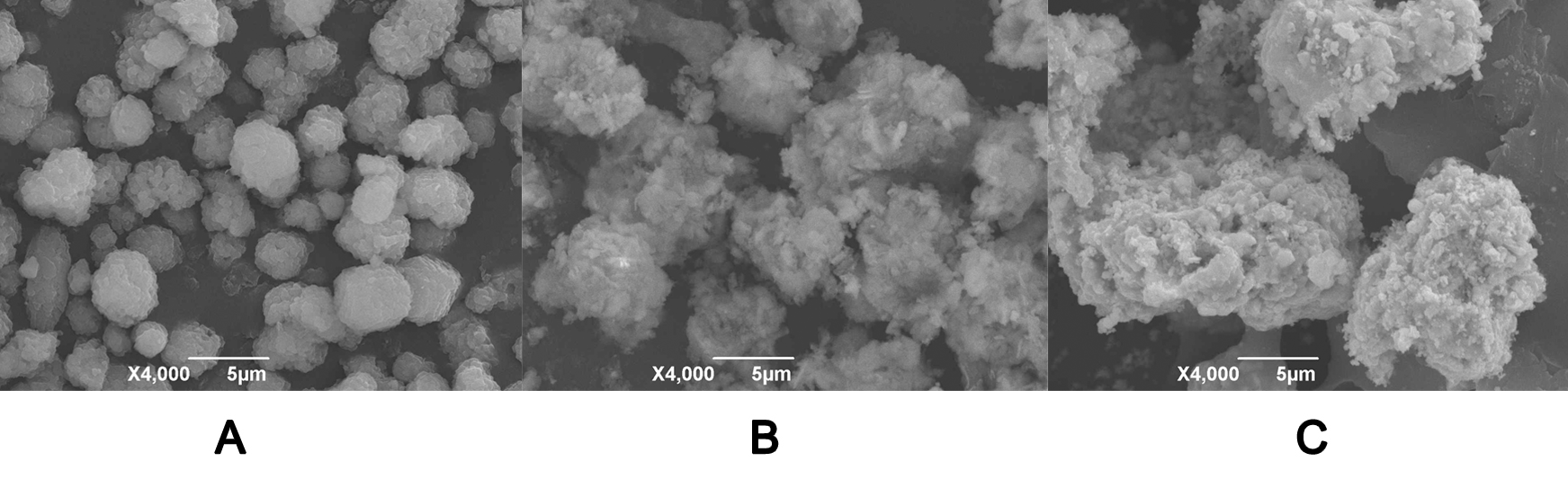

Supplement: Figure S3 — The SEM images of BMO. The SEM images of BMO without adsorbed Ag+ (A), after adsorbing 20 mmol/L Ag+ (B), and after adsorbing 100 mmol/L Ag+ (C). (TIF) [file pone.0081627.s003.tif]
